# What needs to happen to ‘level up’ public health?

**DOI:** 10.1080/21582041.2023.2232765

**Published:** 2023-07-10

**Authors:** Sarah Ayres, Andrew Barnfield, Geoff Bates, Anna Le Gouais, Nick Pearce

**Affiliations:** a School for Policy Studies, University of Bristol, Bristol, UK; b Institute for Policy Research, University of Bath, Bath, UK; c Bristol Medical School, University of Bristol, Bristol, UK

**Keywords:** Levelling up, public health, health prevention, devolution, inequalities

## Abstract

The aim of this article is to examine what needs to happen in central, sub-regional and local government to ‘level up’ public health in the United Kingdom (UK). The Government’s recent *Levelling Up* White Paper outlined ambitious targets for reducing regional disparities, including a ‘mission’ to tackle inequalities in healthy life expectancy and reduce inequalities in the social determinants of health outcomes. However, the approach has been criticised for failing to integrate population health policy objectives, programmes and interventions into the implementation of the levelling up agenda and its associated ‘missions’. Drawing on a case study of promoting healthy urban development in the UK, we examine how the wider determinants of health might be incorporated into the Government’s levelling up strategy. Based on in-depth interviews with 132 urban development actors, our findings reveal that long-term investment in healthy urban development could play a key role in levelling up public health but is not currently part of the Government’s plans. We make a timely contribution to the levelling up debate by placing public health centre stage in social science debates. We conclude by offering a series of recommendations for transformative policy change to level up health.

## Introduction

The aim of this article is to examine what needs to happen in central, sub-regional and local government to ‘level up’ public health in the United Kingdom (UK). The debate is set within the context of the UK Government’s *Levelling Up White Paper* (HM Government, 2022), which sets out an ambitious plan to reduce spatial economic and social disparities between rich and poor parts of the UK. The White Paper (HM Government, 2022, p. 1) aims to tackle regional disparities leading to ‘people everywhere living longer and more fulfilling lives and benefiting from sustained rises in living standards and well-being’. It is viewed as a significant document that offers the potential for a radically new approach to reducing geographical economic inequalities (Martin et al., 2022). The White Paper sets out ambitious targets for reducing regional inequalities by 2030, setting out 12 ‘missions’ across a broad range of areas including employment, productivity, investment, education, living standards and health, all of which are associated with the wider determinants of health (Barton & Grant, 2006). These missions are underpinned by £4.8bn of funding, the bulk of which is allocated for infrastructure development. Plans to level up public health include narrowing the gap in healthy life expectancy between the highest and lowest local areas by five years by 2030 and raising overall healthy life expectancy by five years by 2035. It also pledges to ‘improve wellbeing in every area of the UK and close the wellbeing gap between the top performing and other areas by 2030^′^ (HM Government, 2022, xviii).

The ambition to address the UK’s historical problem of spatial disparity has been broadly welcomed by actors in health policy (Carter, 2022), local government (Local Government Association, 2023) and urban economics (Wood & Swift, 2022). However, there has been disquiet about the potential for Government to achieve its aims (Connolly et al., 2021). These concerns relate to the continuation of a top-down approach to policy objectives and funding streams, a lack of clarity in plans for implementation and evaluation and concerns over available data and evidence to support innovative place-based initiatives (Shearer, 2022). There have been more specific concerns about the health and wellbeing targets. For example, the White Paper does not commit substantial new health funding and the Government’s ambitious health missions are to be delivered principally through individual health service interventions: social prescribing, drug treatment, tobacco control, dietary assistance (Ralston et al., 2022). This has been criticised for not focussing on the wider determinants of health, such as well-maintained housing, education and access to green space, which are shown to have a far greater impact on public health outcomes and reducing health inequalities (Ford et al., 2021). The shelving of the Health Disparities White Paper and the proposal for a Major Disease Strategy has been criticised for renewing the focus on medical conditions, which encourages activity and treatment within the NHS, rather than the conditions in which health inequalities take hold (Nightingale & Merrifield, 2023).

This article explores how the Government might address the wider determinants of health in the implementation of its levelling up ambitions. It does so by focussing on one aspect, namely the link between the urban environment and health. Urban environments have a significant impact on health and wellbeing, most commonly through non-communicable diseases (NCDs), such as asthma, mental health, obesity and cancer, which account for 89% of deaths in England (British Medical Association, 2018). This issue is often compounded by geography. In the areas of England with the lowest healthy life expectancy, more than a third of 25 to 64 year olds are economically inactive due to long-term sickness or disability (Office for National Statistics, 2017). Moreover, the cost to the economy of ill health among working-age people is estimated to be £150bn a year (Eaton et al., 2023). Much of the academic commentary and critique on levelling up has focussed on economic productivity as a measure of regional inequalities (Leyshon, 2021). As Tilley et al. (2023, p. 1) note:

Addressing place-based inequality and improving regional governance are key problems in regional policy, with the most pressing and deeply embedded challenge being productivity.

There has been far less attention on plans to level up public health, especially from scholars outside the public health community. This article seeks to redress this balance by examining the political, institutional and evidence requirements to place public health at centre stage of the Government’s levelling up ambition. It draws on the work of Martin et al. (2021) who provide a comprehensive critique of the scale of the regional disparities problem in the UK, the historical challenges of addressing it and suggestions for how it might be resolved. More specifically, Martin et al. (2021, pp. 9–10) identify seven recommendations for successful ‘levelling up’. In this article we use these recommendations as an overarching framework to interrogate the specific challenge of levelling up public health.

Drawing on a large-scale, qualitative and transdisciplinary dataset we explore what would need to happen in central, sub-regional and local government to implement the seven recommendations outlined by Martin et al. (2021). Following this introduction, we set out our review of the literature on spatial disparities, recommendations for transformative change and the opportunities and constraints to level up public health. The following section outlines the methods employed to undertake the study, followed by the analysis of our interviews. We discuss our findings under the seven recommendations offered by Martin et al. (2021) to explore the potential to level up public health. This article makes a timely and important contribution to the academic and policy debate by applying these recommendations specifically to public health. Our central finding is that the Levelling Up White Paper presents an opportunity to tackle health inequalities but not in its current form. Instead, a more radical and innovative approach is required that focuses on long term, preventative measures and the wider determinants of health. We argue that healthy urban development could play a role but is not currently part of the Government’s plans.

The literature review below is structured as follows. First, spatial disparities in the UK are examined and Martin et al.’s (2021) seven recommendations for redressing them introduced. Second, the Government’s Levelling Up White Paper (HM Government, 2022) is introduced with a specific critique of the Government’s mission to level up public health. Third, the potential for a focus on long term, preventative health in examined. More specifically, we explore the wider determinants of health and the link between the urban environment and health outcomes.

### Spatial disparities in the UK and recommendations for transformative change to level up

Despite the attempts of successive UK Governments to rebalance the economy and close regional divides, ‘the UK is still one of the most regionally unequal countries in the developed world’ (Webb et al., 2022, p. 5). These divides are long-standing. Inequality in regional GDP per capita in the UK was broadly the same at the end of the twentieth century as it was at the start (Geary & Stark, 2016). During the period of state-led industrial and manufacturing development between the 1930s and 1970s, regions outside London and the wider South-East of England experienced faster economic growth and regional economic inequality declined. However, this process was reversed by deindustrialisation and neo-liberal globalisation in the 1980s (Geary & Stark, 2016; Hudson, 2016). Since the 1980s, inter-regional inequality in the UK has worsened, driven by the entrenchment of a highly financialised, consumption-led growth regime (Hassel & Palier, 2021). The UK is now the most spatially unbalanced large, advanced Organisation for Economic Cooperation and Development (OECD) economy (McCann, 2019).

Scholars and public policy commentators have long ascribed the persistence of the UK’s regional disparities to its highly centralised system of governance (Carrascal-Incera et al., 2020; Raikes et al., 2019). Ayres (2022), for example, notes that the UK is one of the most centralised countries of its size in the developed world and English local government has the most circumscribed powers of any equivalent tier internationally. These challenges have made it difficult to address spatial disparities at the appropriate level of governance. In recent years, ‘left behind places’ is a term used to describe localities with a broad variety of disadvantage (Leyshon, 2021). In the UK, outside of London, cities and large towns of the midlands and northern England have been highlighted as ‘left behind’ places (MacKinnon et al., 2022). They are places that have been adversely affected by austerity, globalisation, economic and technological change (Pike et al., 2023). Key features include economic (e.g. unequal access to jobs, income and opportunity), material (e.g. deficiency of infrastructures and services), and social factors (e.g. sense of cultural, political disaffection and marginalisation) (Rodriguiz-Pose, 2018; Rodríguez-Pose et al., 2023).

Health is one key area with substantial inequalities linked to spatial disparities. In England there are large and increasing differences in life expectancy between and within regions (Bambra, 2016; Marmot et al., 2020). This is strongly linked with deprivation. For example, life expectancy of those living in the most deprived compared to the least deprived areas is 9.7 years fewer for males and 7.9 years for females (Office for National Statistics, 2022). Inequalities in healthy life expectancy (an estimate of years spent in good health) are even greater with around 20 years fewer of healthy life years for those in the most deprived areas (ibid). The differences in health outcomes across the country are long-established but have been exacerbated by the COVID-19 pandemic. Indeed, COVID-19 exposed health disparities within and between places (Hambleton, 2020) and drew specific attention to the links between the urban environment and health (Marmot, 2022).

Health inequalities from non-communicable diseases, such as asthma, mental health, obesity and cancer (Eaton et al., 2023), are associated with a range of social determinants, including the places that people live (Bambra, 2019). The World Health Organisation estimates that ‘one in eight deaths is linked to air pollution exposure – mostly from heart and lung disease, and stroke’ (World Health Organisation, 2015, p. 1). This is particularly a risk in urban areas where people living in more deprived neighbourhoods are exposed to greater environmental and social risk factors such as poor-quality housing, higher levels of air pollution, worse transport connectivity and access to fewer and worse quality green spaces (Borrell et al., 2013; Carmichael et al., 2019; Geary et al., 2021).

Martin et al. (2021, p. 108) argue that ‘the performance of left behind places has progressively fallen away from the national average … and has become spatially and systematically entrenched’. Moreover, the scale and nature of UK geographical inequality means that a transformational shift in policy and resource commitment are required to achieve the levelling up ambition. Shortly before the formal publication of the *Levelling Up White Paper* (HM Government, 2022), Martin et al. (2021) identified seven recommendations to achieve the Government’s levelling up ambition. These recommendations advocate strongly for the redistribution of resources, rebalancing of the UK economy and devolution to localities as the solution. However, it is important to note that this position is not shared by all commentators in the social sciences. For example, Hooghe et al. (2016) note that while the direction of change towards global decentralisation is clear, the pattern has not been uniform. They contend that devolution takes many forms and there is no clear and consistent evidence on the link between decentralisation and policy effectiveness. Likewise, Bucek and Ryder (2015, p. 2) argue that in the absence of concrete evidence on the efficacy of devolution, the global trend towards decentralisation ‘may be one of the greatest public administration experiments ever undertaken’. There are also those who do not believe that Government attempts to rebalance the UK economy through policy interventions can compete with global economic forces (Ehrlich & Overman, 2020). Instead, they advocate for clustering of economic prosperity and productivity. Despite these differences in academic perspective, Martin et al’s seven recommendations offer a suitable framework for interrogating plans to level up public health as they relate to the key themes in the Levelling Up White Paper. Each is discussed in turn below.

### Grasp the transformative moment for local, regional and urban development policy

A series of extreme events have created an unprecedented set of factors for Governments around the world to contend with. Since Martin et al. (2021) published their recommendations, the Ukraine war and rising living costs add to the economic challenges facing the UK government from Brexit and the aftermath of the COVID-19 pandemic. In the UK, governing in ‘turbulent times’ (Ansell et al., 2021) has raised challenges but also opportunities for transformative change. The need for a radical step change is clear and pressing. ‘Relying on existing approaches risks reproducing the problems of merely ameliorating rather than resolving spatial inequalities and addressing their symptoms rather than underlying causes’ (Martin et al., 2021, p. 112). Wills (2016) notes that the inherent propensity for top-down elite control in British politics undermines our ability to imagine other ways of organising the state. One way of addressing this is to develop new and bold ‘imaginaries’ about political decision-making to tackle complex social problems (Healey, 2018).

### Establish a clear and binding national mission for ‘levelling up’

There have been accusations that levelling up is the latest in a list of politically useful but empty slogans, used as a substitute for real devolution of power and resource (Fransham et al., 2022). One way of addressing this is to articulate levelling up as ‘a clear, explicit and binding national mission, akin to its net zero carbon commitment, and with a legally binding underpinning’ (Martin et al., 2021, p. 114). This move would shift ‘levelling up’ from a political slogan to a core mission. Shearer (2022) also underscores the need for clear and realistic targets, performance indicators and supporting metrics so that Government can be held to account in key areas associated with levelling up. Combined Authority Mayors and civic and business leaders in the North of England have gone further and called for constitutionally guaranteed commitments to reducing regional disparities (Greater Manchester Combined Authority, 2023).

### Realise the potential of place in policymaking

Local context is fundamental to understanding the lived experience of people in distinct places (Rodríguez-Pose & Storper, 2020). Local knowledge and expertise provide an understanding of what it means to be ‘left behind’ and can form the basis for place-based initiatives (Chyn & Katz, 2021). However, meaningful decentralisation of powers and resources are integral to enabling local discretion and sensitivity to local contexts and needs. ‘It is critical to involve not just local government but also local community-based organisations and third sector bodies in meaningful dialogue in the local economic development agenda, building on community-based assets and developing local skills’ (Martin et al., 2021, p. 118).

### Decentralise and devolve towards a multilevel federal polity

Much academic research has principally classified left behind places in relation to poverty or economic performance (McCann et al., 2023; Tilley et al., 2023; Pike et al., 2018). There is far less evidence on the important factors outside of economics that have a compounding effect on spatial inequalities (Kemeny & Storper, 2020). More recently, however, there is growing recognition of the need to engage with disadvantage and geographical inequalities from a transdisciplinary perspective (Black et al., 2018). For example, Houlden et al. (2022) argue that understanding left behind places requires an appreciation of economic, political, health, social, environmental, and infrastructural aspects. This type of thinking represents a ‘systems approach’ to problem solving and policy making (Colander & Kupers, 2014). Levelling up requires a shift towards a more coherent and integrated governance system in England that can accommodate a systems approach to policy making (Martin et al., 2021). This does not require a federal polity per se, but rather the decentralisation and devolution of policy responsibilities and resources to enable integrated, systemic policymaking at sub-regional and local levels in England.

### Strengthen subnational funding and financing

Successful devolution requires flexibility in local powers and control over resources. ‘Multi-year and flexible financial settlements enable subnational actors to formulate strategic, long-term and more transformational plans with appropriate funding and financing’ (Martin et al., 2021, p. 121). Indeed, Tomaney and Pike (2020) argue that addressing left behind places will require a radical devolution of power and resources alongside the rebuilding of local government which has been considerably reduced since 2010. Central government’s austerity drive saw funding to local government reduced by 50% between 2010 and 2011 and 2020 and 2021, with the poorest areas most negatively affected (Alexiou et al., 2021).

### Embed geography in the national state and in national policy machinery

The past few decades have been characterised by numerous reforms and policies aimed at decentralising powers and responsibilities to the subnational tier. However, ‘the system and culture of central government has been too slow to adapt to the changing structures of subnational administration’ (Newman & Kenny, 2023, p. 6). Central government has been unable to grasp the potential for place-sensitive policies, therefore undermining the potential of local decision making. Instead, Martin et al. (2021, p. 125) suggest the ‘aim should be to enable and deliver better alignment vertically between mainstream national and subnational policies and expenditure, and horizontally at each level through collaboration between public, private and civic actors’. This involves joining up key policy areas, such as education, health, housing and infrastructure and ensuring that left behind places have adequate resources commensurate with their needs.

### Improve subnational strategic research, intelligence, monitoring and evaluation capacity

Actors who shape and inform policy agendas and debates commonly use evidence as part of their efforts to reach decision makers (Bates, Ayres, et al., 2023). Evidence and metrics are central to decisions over what can be devolved and how local leaders can be held to account for performance (Sandford, 2022a). However, ‘developing an evidence-led approach is a long-term project; demand for evidence needs to be created; local partners need to be involved; external challenge should be encouraged; negative findings need to be acknowledged and addressed; compelling narratives need to be created built upon the evidence; evidence needs to be utilised and acted upon; and the development of evidence needs to focus upon long-term drivers of economic growth’ (Martin et al., 2021, p. 127).

### Levelling up public health

Published in February 2022, the *Levelling up White Paper* (HM Government, 2022) is the Government’s flagship project to reduce regional inequality and enhance opportunities for all. It is intended to remedy entrenched spatial inequalities and the plight of left behind places by addressing many of the administrative barriers identified in the previous section. It sets out 12 ‘missions’ (Table 1) to be achieved by 2030 across a range of areas from productivity to health. The missions take a cross-cutting approach and covers many areas such as living standards, transport, education and skills, all of which are associated with the wider determinants of health.

The White Paper acknowledges that past attempts at regional rebalancing have failed due to a top-down, siloed approach to target setting and implementation. Instead, the 12 missions are intended to play a coordinating role by promoting a ‘whole system’ approach that acknowledges the links between different domains and parts of government. Overall, the ‘concept of levelling up enjoys widespread support’ in policy and academic domains (Connolly et al., 2021, p. 523). However, there have been concerns that levels of funding and allocated powers to localities are insufficient to address the scale of the problem (Overman, 2022; Arnold & Hickson, 2022).

Likewise, the health and well-being missions in the White Paper have been praised for their ambition and for recognising the importance of health determinants (Shearer, 2022; Arnold & Hickson, 2022; Thornton, 2022). However, these goals have been criticised for not setting out the plans necessary to address health inequalities. Austerity-era cuts to healthcare and prevention services, and the public health grant, have worsened existing health inequalities (Ralston et al., 2022; Merrifield & Nightingale, 2021) and current funding commitments in the levelling up agenda do not do enough to overcome these cuts (Lacobucci, 2022).

While there is a lack of co-ordination across policy departments in the UK government (Coyle & Muhtar, 2023), levelling up presents an opportunity to integrate health across sectors and government departments. For example, the White Paper acknowledges that ‘successful local growth policy requires strategic coordination across the different arms of policy, including transport, skills, health, business, finance, education, and infrastructure’ (HM Government, 2022, p. 100). While there is a central focus on economic growth, the descriptions of multiple factors responsible for widening geographic differences in the UK indicates a willingness in Government to think about the problem from a holistic, systems perspective. Currently, the Government’s plans for achieving the two health and wellbeing missions (Table 1) focus on improvements to service delivery. However, 80% of what causes ill health is determined outside the health sector (Taylor, 2022). Some health outcomes are associated with other missions in the White Paper, such as those related to employment, community, quality of rental housing and crime. However, population health policy objectives, programmes and interventions are not integrated into the wider levelling up agenda and its associated ‘missions’. Ogden et al. (2022) argue that any meaningful improvement to health outcomes and inequalities needs to address the wider determinants of health by prioritising the root causes of ill health and inequality. Systemic, population-level interventions will have more impact on increasing life expectancy than relying on individual-level interventions to bring about change, as the White Paper does (Dixon & Everest, 2021). While the complexity and uncertainty of tackling the wider determinants of health is acknowledged (Greer et al., 2022), there has been widespread criticism about a lack of focus on preventative health measures in the levelling up strategy (Alexiou et al., 2021).

### Promoting long-term, preventative health through healthy urban development

Despite compelling evidence on the links between urban environments and health inequalities, there has been little progress on tackling (un)healthy urban development (Giles-Corti et al., 2022). This is partly because the resources required to address the problem are dispersed across many agents (Black et al., 2021). Those with the power to act, such as private developers and investors, do not see health as their concern. This challenge is highlighted by Greer et al. (2022, p. 718) who acknowledge that ‘everything affects health, but not everybody thinks health is their problem’. Despite this challenge, there is growing recognition that levelling up health will only succeed if there is collaboration across the whole of government (Health Foundation, 2023; Meier et al., 2023) along with re-investment in local government and places with the greatest health need (Alexiou et al., 2021).

Currently, tackling the wider determinants of health, such as improving urban environments, does not feature in the Government’s levelling up plans. Focus needs to be on low agency and upstream policy interventions that target structural factors to improve health, ‘rather than requiring individuals to invest a high degree of their own resources or effort to benefit’ (Ford et al., 2021, p. 26). Instead, the Government’s strategy to level up health is centred on high-agency interventions such as ‘supporting people to change their food and diet, and tackling diagnostic backlogs’ (HM Government, 2022, p. 422). Addressing the wider determinants of health would require a reimagination (Healey, 2018) of the Government’s strategy to level up health. This would involve thinking more creatively and imaginatively about long-term, preventative health.

## Methods

The findings in this article draw on research conducted as part of a large UK Government-funded research grant, ‘Tackling the root causes upstream of unhealthy urban development’ (TRUUD), 2019–2024, £6.7 m, funded by the UK Prevention Research Partnership. The study explored decision making in England’s urban development system and the factors affecting how health is included. A detailed description of the study methodology is provided by Bates, Le Gouais, et al. (2023). The data in this article emanates from seven data gathering teams (Table 2).

Stage one of the research involved mapping the urban development system using a qualitative approach, facilitated by in-depth semi-structured interviews with critical actors. A purposive sample was informed by desk-based searches, a policy review, established professional contacts and snowballing. These activities generated a database of approximately 500 urban development stakeholders operating at a global, national and local level. Some stakeholders were selected due to their expertise and experience in major subsystems of urban development such as property development, transport systems and land acquisition. Others had expertise in cross-cutting areas such as policymaking, law, sustainability and finance. To refine the sample further, while maintaining the breadth of the sample, the team identified two criteria for selection (i) high levels of influence over decision making and (ii) actor’s in-depth knowledge of the system.

The design of interview questions was guided by a broader set of research questions agreed by the whole team to investigate decision-making in urban development (Appendix A). Interview questions were tailored by individual data gathering teams appropriate to each sector but were discussed extensively across the teams to ensure complementarity and the ability to triangulate data. 132 participants were interviewed across seven data gathering teams (Table 3). Semi-structured interviews were conducted on-line between May – September 2021. Interviews lasted on average 55 mins (range 26–112). Interviewees were assured of confidentiality and informed consent was obtained.

The team undertook a multi-stage, transdisciplinary analysis that started in September 2021 and ended December 2021. Transdisciplinary research integrates knowledge across academic disciplines to create a comprehensive approach (Simon et al., 2018). Coding involved a deductive and inductive process (Clarke & Braun, 2021). Deductive codes were identified through concepts in the literature and the research questions and inductive codes were added during analysis. Through this process the team developed a large coding framework in NVivo12 (a commonly used computer-assisted qualitative data analysis software), with over 300 individual codes grouped into 23 overarching categories. The coding categories can be seen in Appendix B. After coding, each of the seven data gathering teams summarised their own data within each of the 23 categories. The team then split into a series of sub-groups to analyse data from across all seven data gathering teams. Patterns and observations from across the entire dataset were extensively discussed as a transdisciplinary team to elicit findings.

An early output of this transdisciplinary analysis was a high-level summary overview of study findings to help the team understand decision-making in the urban development system. Patterns included similarities and differences between stakeholders relating to, for example, power and control in the system, the relationships between local and central government and commercial determinants on urban development. The findings in this article emanate from the high-level summary overview. However, to get a deeper understanding of the data in the context of the levelling-up agenda, and to identify illustrative quotes, we also drew on data from the individual team summaries. We applied our data to Martin et al.’s (2021) seven recommendations for successful levelling up to help understand what changes are needed for the development of urban environments that can help to level up health in the UK. We explored the current barriers to promoting healthy urban development, what would need to change in national and local government to enable the pursuit of more innovative and creative approaches to preventative health and the evidence requirements of actors to be able to make brave and bold decisions on tackling the wider determinants of health associated with the built environment. We supplemented this analysis with insights from the literature commentating on the White Paper. We analysed academic and policy documents that review and reflect on the government’s plans for levelling up and the implications of this for health. We include findings from this literature alongside our interview data in our analysis.

This methodology has several acknowledged strengths and weaknesses. First, regards its strength, large scale, transdisciplinary, qualitative interviewing is relatively rare due to the extensive resource and disciplinary expertise required. The research emanating from this project offers a unique and timely opportunity to explore urban development decision-making from many different perspectives. Second, interviews have been conducted with a large range of actors at the heart of the UK urban development process. Their diversity of experience across the complex system of urban development decision-making strengthened this exploratory study. Access to these respondents was possible through the established professional contacts of team members. Third, this study adopts an in-depth qualitative methodology aimed at providing critical insights into the day-to-day realities guiding decision making (Rhodes, 2013). There are, however, some acknowledged weaknesses. Undertaking transdisciplinary, team coding of a large dataset is a challenging task with different expectations and interpretations of codes and data across the team. We engaged in extensive team coding discussions, the careful design of a team NVivo code book and intermittent analysis of team coding practices to mitigate this risk and support the development of a robust transdisciplinary data set (Bates, Le Gouais, et al., 2023). The interviews took place in the first year of the pandemic when attention for many policy actors was focused on COVID-19 and the narratives on urban development and health that were enhanced at this time. The White Paper had not been released when we carried out these interviews. Therefore, we did not ask our participants specifically about their reactions to the government’s levelling up missions, how these could be achieved or what additional actions were needed to level up public health. However, the Levelling Up agenda was well-established by this time having been a key theme of the Conservative Party’s 2019 election manifesto. A ‘Levelling Up Fund’ worth £4.8 billion had been announced in November 2020 to support urban regeneration and infrastructure projects as part of a Spending Review that set out the intention to ‘put levelling up at the heart of policy making’ (HM Treasury, 2020, p. 2). The fund and wider Levelling Up agenda were therefore highly relevant to many of the urban development stakeholders we interviewed.

### Analysis and findings: what needs to happen in central, sub-national and local government to level up health?

In our data analysis, we have applied Martin et al.’s (2021) seven policy recommendations for tackling spatial disparities specifically to the Government’s ambition to level up public health. This has resulted in the development of our own recommendations for tackling health inequalities as part of the levelling up agenda (Table 4). Each of these is discussed in turn below.

#### Grasp the transformative moment for local, regional and urban development policy

Levelling up health requires a major shift towards a long-term commitment to health prevention and tackling the wider determinants of health, including developing healthier urban environments. The need for ‘a radical step change’ (Martin et al., 2021, p. 112) to address the underlying causes of inequalities remains pressing at a time of significant economic pressure. In addition to the financial burden of COVID-19, the UK is experiencing the ongoing impacts of Brexit and the war in Ukraine with rising energy prices and inflation, and the healthcare system is under severe pressure with long waiting lists and staff shortages (Nacer & McKee, 2023).

While this creates obvious challenges for increasing spending, it highlights the economic case for investing in a healthier society. This builds on narratives from the COVID-19 pandemic that demonstrate the need to take a more comprehensive approach to understand the impacts of public health investment, as a public health official explained, ‘COVID will absolutely crystalise that you can’t have good health without the economy, and you can’t have a good economy without health’. Long-term illnesses, including the effects of COVID-19, and the struggling healthcare system are cited as key reasons for high numbers of people leaving the workforce in the UK (McKee, 2022). Staffing shortages highlight the wider consequences of poor health and the economic benefits of health creation. This resonates across the urban development system. A real estate consultant explained that from an employer’s perspective, ‘if they have a happy, healthier workforce that then does actually covert into better work essentially, and a more productive team’. Investing in tackling the wider determinants of health will support the development of a healthier workforce and a more resilient society, which will benefit the economy and reduce pressure on the healthcare system. It will help to achieve Mission 1 in the White Paper to increase pay, employment and productivity throughout the country by 2030 (HM Government, 2022) as a healthier workforce means a larger and more productive supply of labour due to reduced short- and long-term absence from the workforce caused by sickness and caring responsibilities and improved productivity while at work (British Medical Association, 2022; Haldane & Rebolledo, 2022).

Recommendations to government in the Hewitt Review (Hewitt, 2023) call for a reimagination (Healey, 2018) in focus from treating illness to promoting health, including increasing funding for prevention. The review states that ‘shifting the focus upstream is essential for improving population health and reducing pressure on our health and care system’ (ibid, 7). Going beyond rhetoric about the importance of prevention and to make long-term funding commitments, including multi-year departmental allocations, is key. Delivering the investment required would represent a change in direction that has seen the public health grant for local authorities cut by one quarter since 2016 (Finch & Vriend, 2023). It will require investing in long-term benefits in a system characterised by short-termism. A local authority property officer illustrated the point, ‘there are savings targets that have to be met today, which always trump tomorrow because the implications of not meeting savings targets to balance the books today are significant’. Like-wise, a scientific advisor to government described the pressure to cut spending and adhere to short-term political cycles, ‘we elect potentially a new government both in our cities and nationally every four or five years, so these things take decades, and I keep saying we need very brave politicians if we’re going to get to where we need to be’. Transformative change will require ambition and bravery amongst political leaders who are operating in turbulent times (Ansell et al., 2021).

#### Establish a clear and binding national mission for levelling up

The focus on addressing wider determinants and prevention must be driven as a priority from the centre of government. Key Whitehall decision-makers from departments that shape urban development and health should be brought together with an agenda to embed health targets across government, reporting to the Prime Minister. Our findings show that health is not currently a key priority for urban development policy actors. The links between urban policy objectives and health outcomes are recognised. However, currently, health is less influential than the delivery of other established urban policy targets such as numbers of new homes, or cross-cutting issues such as the Government’s net zero and productivity agendas. A Whitehall civil servant in a cross-department team explained,

when you look at some of the narrative around levelling up, it’s much more about productivity and average incomes … I think it [health] is in there in the narrative about healthier environments and healthier people, but it comes after the productivity stuff. I’d say that’s probably quite reflective of how central government thinks.

A barrier to action is government silos (Shearer, 2022). Policy teams and departments have narrowly conceived goals and there is a lack of joined up working between health stakeholders and those working on delivering urban policies at national and local levels. This is reflected across government as ‘the institutional structure for collaboration and co-ordination is inherently absent and depends on buy-in across senior politicians’ (Coyle & Muhtar, 2023, p. 7). A local politician explained that ‘people still very much work in silos and do their own little bit’. The consequences are that preventing NCDs is not seen as the responsibility of urban *or* health policy makers (Greer et al., 2022). A government scientific advisor stated that, ‘the challenge is that trying to address the social determinants of health is very difficult when it falls outside the responsibility of the minister that we are ultimately working for and are accountable to’.

A clear steer from central government is needed to prioritise the wider determinants of health across departments. Importantly, it must integrate health actors and agendas into other policy areas so that addressing health inequalities becomes the responsibility of departments who together shape urban development. This argument was made by a senior health official,

We need a Whitehall narrative on this … we need the Department of Health and Social Care to be at the same table as MHCLG [Ministry for Housing Communities and Local Government - the former Levelling Up Department], the treasury, cabinet office.

To move beyond ‘empty slogans’ (Fransham et al., 2022, p. 1) a commitment to reducing health inequalities requires ownership from the top of government. This could be achieved by establishing a ‘cross-ministerial board with teeth, for example, reporting directly to the Prime Minister, attended by secretaries of state and with a secretariat provided by the Cabinet Office to act as a broker across government. The programme should be firmly linked to the levelling up agenda’ (Dixon & Everest, 2021, p. 12). The mission needs to be articulated in key national urban development policies to reinforce the message and to support local authorities to act, as a local authority urban planning officer expressed, ‘I think the key would be to get to a point that things were tangibly and specifically embedded through national policy in a meaningful way’. This view was also supported by a local authority public health official who agreed that ‘in the national planning policy framework, or whatever replaces that, and in the regional and local plans, we need to have clear endorsement of the need for radical change’.

#### Realise the potential of place in policymaking

Levelling up needs to accommodate the specific needs and circumstances of localities so that contextually relevant, place-based solutions can be developed (Chyn & Katz, 2021). Top-down targeting of resources and solutions can miss important contextual understandings about how to address local heath inequalities. The White Paper (HM Government, 2022, xiii) pledges to ensure that ‘levelling up is not directed from London … and that Government is taking decision-making closer to the communities’. Local Government will need to work with the public, private and third sector organisations to ensure that affected publics are engaged in ‘meaningful dialogue’ on localised problems and what can be done to address them (Martin et al., 2021, p. 118).

A local authority property official underscored this point by describing public knowledge as ‘*understanding customer intelligence’* in planning. Likewise, a local authority transport official indicated ‘*we need to see them [citizens] as allies because at the end of the day we both want the local environment to improve … they’re our eyes and ears … sort of local geographers on which we rely’*. There are, however, challenges in enabling meaningful community engagement. The lack of specific engagement requirements for urban development, beyond statutory consultations (HM Government, 2015) can result in community views being expressed too late to be impactful. A local authority engagement official explained the predicament,

the involvement of communities is very planning-led, so communities are involved at the point of the formal consultation window. All the decisions have been made by that point really, and it’s a kind of: ‘we’re consulting you on what we’re about to do and we may or may not tweak some elements as a result’.

This suggests that the current system needs changing so that public voices are heard earlier. The process needs to ensure that engagement goes beyond ‘tick box’ approaches, which are unlikely to be useful. Enabling meaningful engagement with people living in left behind communities, who are sometimes described as ‘hard to reach’ (Towns Fund, 2020), can be particularly difficult, as a local authority transport official explained, ‘you hear the loudest people, that doesn’t necessarily mean they’re the correct people’. Local government needs to be resourced properly to develop an understanding of complex local health needs (Alexiou et al., 2021). Moreover, there needs to be clearer requirements for early engagement by developers within the planning system. This could be included in local plans, which set out local authority planning policies (Planning Inspectorate, 2022), and/or central government’s National Planning Policy Framework (Ministry of Housing, Communities and Local Government, 2021). Central and local government need to fully recognise the benefits of meaningful engagement (Brownhill & Parker, 2010) and allocate the requisite resource so that investment strategies can be properly informed by local priorities and need.

A fine-grained understanding of local context provides an opportunity to identify otherwise hidden inequalities within local authority areas. The Levelling Up White Paper states, ‘By 2030, the gap in Healthy Life Expectancy (HLE) between local areas where it is highest and lowest will have narrowed’ (HM Government, 2022, iii). However, many neighbourhoods with the worst health outcomes are not in the bottom 10% of economically deprived local authority areas (Health Foundation, 2022). Resources to level up public health need to go to those places with the greatest health needs, through targeted investment informed by local knowledge and data.

#### Decentralise and devolve towards a multilevel federal polity

Programmes that target the specific needs of local areas are more likely to be effective at reducing inequalities (Davey et al., 2022). Local and regional variations, such as population demographics and health outcomes, mean that a ‘one size fits all’ approach is frequently inadequate. Instead, reducing gaps between areas in Healthy Life Expectancy will require targeted approaches that address local priorities, particularly focused on areas with worse health outcomes (Ford et al., 2021). This requires further devolution of powers to localities and sub-regional combined authorities over urban development and simplified funding mechanisms that give greater flexibility in how resources from central government are used, such as in single settlements announced as part of the new ‘trailblazer’ deals in two of England’s ten combined authorities (Department for Levelling Up, Housing and Communities, 2023).

In England urban development is shaped largely by national priorities and funding. Our findings highlight how targets set by national government can restrict an innovative, ‘systems approach to policy making’ (Martin et al., 2021, p. 120). For example, national house building targets were commonly criticised as a driver of decision-making over quality of place and the needs of local communities, as a local authority planner explained,

It should be place making at the heart of it and not led by formulaic numbers which are derived from a formula to achieve a notional national target which is further and further away from need.

A scientific advisor to Government on health indicated that local actors lack the power and resources to respond to local priorities, ‘the power isn’t in the local authority to change some of the things that matter, the things that are determining that agenda locally, whether that be planning, regulation, or housing stock’. Further devolution of powers and resources to localities would allow the local autonomy to set targets and take actions tailored to specific communities (Houlden et al., 2022). For example, there is evidence that devolved powers over local services in Greater Manchester have been associated with increased life expectancy, with the greatest effects in areas of higher deprivation (Britteon et al., 2022).

Delivered as part of the levelling-up agenda, the new ‘trailblazer’ deals for Combined Authorities in Greater Manchester and the West Midlands (Department for Levelling Up, Housing and Communities, 2023) demonstrate how greater powers and resources can be devolved. Both city-regions gained new responsibilities, including the delivery of affordable homes and retrofitting houses along with enhanced mayoral powers including strategic leadership on housing (Sandford, 2023). For example, in Greater Manchester ‘the government will devolve £150 million brownfield funding to Greater Manchester Combined Authority to deploy across the region to drive placemaking, housing, commercial development and urban regeneration’ (HM Government and GMCA, 2023, p. 7).

Current funding mechanisms are fragmented and include many separate funding pots, which restricts autonomy over spending and are potentially reformed through the trail-blazer deals. A transport planner in a Combined Authority indicated that, ‘the ideal situation is that (the city) has funding for all these different areas and then decides how to spend it and then you can get some better cross funding at the city region level around health and transport investments’. Under the ‘trailblazer’ deals, a single funding settlement will cover an entire Spending Review period, providing greater flexibility for spending to address inequalities within the Combined Authorities (Henderson et al., 2023). In addition, the West Midlands Combined Authority is to be given a new formal duty to improve the public’s health and the UK government has committed to considering ‘relevant new future, funding streams relating to population health improvement and prevention’ as part of the department-style single settlement (HM Government and West Midlands Combined Authority, 2023, pp. 67–68). These deals provide models for the types of changes required to tackle the wider determinants of health in England that can be built upon for a more radical extension of devolution to Mayoral Combined Authorities.

#### Strengthen subnational funding and financing

Tackling the wider determinants of health requires re-thinking funding models to encourage preventative health (Barry, 2021). The Levelling Up Fund is yet another example of centrally administered competitive, short-term, bid-based funding that undermines local authorities’ ability to act collaboratively and innovatively to solve complex problems. This type of funding places limitations and assurance requirements on the local applicant, including the requirements of a business case, mandatory reporting and evaluation. These stipulations restrict the development of place-sensitive, ‘transformational plans’ (Martin et al., 2021, p. 121) as central government mandates may not align with local preferences (Sandford, 2022b). Martin et al. (2021, p. 123) suggest that what is required is a ‘move away from the model of local actors being forced to make ongoing competitive bids to national government centres. Such approaches reinforce centralised and top-down devised initiatives and criteria, further favour places that are already the best equipped with capacity and resources and constitute “strategies for waste” through duplicated and wasted effort’.

Our data shows that bid-based pots of national funding have a determinantal effect that undermines the ability of local government to prioritise health. Central government funds come with imposed top-down conditions and have largely replaced direct local government funding. This limits discretion for local innovation (Tomaney & Pike, 2020). A senior Whitehall official in DLUHC explained that bid-based funding,

somewhere in the region of £20 billion [is distributed] through the Ministry for things such as homelessness, cladding, town centre renewal and regeneration, monies towards the new settlements and such like. But a lot of that has to be bid for and is monies that perhaps 15 years ago would have gone straight to councils and be spent by councils. But is now much more centralised as less monies are pushed out to local government.

One remedy is to extend the offer of a ‘single pot’ of money as offered in the new trail-blazer deals outlined above. Alongside public spending, further devolution of tax-raising powers, levies and precepts or tax revenue retention to Mayoral Combined Authorities would also enhance sub-national policymaking and the ability of local leaders to invest in place-based, preventative policies. At the national level, another option is to include health outcomes as a strategic objective in central government funding priorities and guidance. For example, the Levelling Up Fund could include an incentive to incorporate health outcomes and inequalities in local investment decisions for infrastructure development. Embedding health criteria into the funding priorities, guidance and evaluation of the Levelling Up Fund would provide a clear signal that health outcomes are central to the Government’s levelling up mission and should be thought about when considering wider investment in infrastructure (Brand-Correa et al., 2022).

#### Embed geography in the national state and in national policy machinery

Martin et al. (2021) make several bold suggestions for promoting place-sensitivity in central government. Some of these suggestions have been met, including, a new Minister and Department with a specific remit for Levelling Up. There has, however, been an element of instability in leadership, caused by three changes in the Secretary of State for Levelling Up since 2019. Despite this, the Department for Levelling Up, Housing and Communities (DLUHC) has a natural remit for policies associated with devolution, infrastructure, housing and communities. These are policy areas with a clear line of accountability to the Minister and Department responsible for levelling up. However, responsibility for levelling up public health rests with the Department of Health and Social Care (DHSC), which is predominantly delivery focused. As such, a focus on health in policy areas outside of DHSC that are important health determinants currently falls between the cracks of Whitehall administrative structures and accountabilities for levelling up. We advocate for closer working between the DLUHC and DHSC if preventative health is to be prioritised in policy areas with health impacts.

A lack of clear accountability nationally filters down to the local level. The Levelling Up White Paper outlines increased forms of local accountability for sub-national governance. More specifically,

the UK Government will introduce a statutory obligation to report annually on progress towards meeting the Levelling Up missions. The report will draw on the metrics set out in this White Paper and provide rigorous analysis and monitoring of progress in reducing regional disparities. (HM Government, 2022, p. 156).

This involves the creation of a new accountability framework which includes ‘clear roles and metrics for assessment, strong local scrutiny mechanisms’ and ‘appropriate forums for local media, local councillors and local residents to review the performance of authorities with devolved functions’ (HM Government, 2022, p. 139). The commitment also includes establishing an independent public body to publish data for sub-national geographies, creating a ‘spatial data unit’ within DLUHC and publishing evaluations of place-based policies (Sandford, 2022b).

However, this accountability framework and reporting is likely to focus on those areas that can be easily identified, quantified and evaluated and may not be appropriate for cross-cutting issues such as healthy urban development. A senior local authority leader described the challenge of combining data sets around complex, cross-cutting governance agendas,

Issues around data quality, security, data sharing between partners outside a [policy] area, just the complexities of inclusion of different business systems and getting them into the data area is a barrier. Barrier after barrier.

The absence of clear accountability frameworks and the problems of reliable and accessible data need to be addressed if the wider determinants of health, linked to the urban environment, are to be included. Bambra et al. (2010, p. 284) argues that the focus on improvements to health service delivery may reflect ‘that lifestyle issues are often easier to identify and treat … with evidence on tackling the wider social determinants being less apparent and less accessible to policy makers and practitioners’. Indeed, the complexity and uncertainty of holding central and local actors to account for the wider determinants of health are barriers to transformative change that need to be overcome.

#### Improve subnational strategic research, intelligence, monitoring and evaluation capacity

Evidence-informed policy is often portrayed as the norm. Yet, within a complex system there are multiple other influences on decision-making which may be value-driven and political (Bates, Ayres, et al., 2023). For example, Andy Street, the West Midlands Combined Authority Mayor, complained that levelling up funding was not being devolved for local decision-makers to decide how best to spend it (Street, 2023). Rather, decisions were taken by Whitehall civil servants and conservative politicians looking to cement recent political gains (Diamond et al., 2023). Despite this, evidence is an important part of the decision-making process. Many types of evidence are used by policymakers to inform urban development decisions, including population data, public opinion and health inequality data, such as the Index of Multiple Deprivation (Ministry of Housing, Communities and Local Government, 2019). Callway et al. (2023) argue that local health data needs to be used more effectively to influence investment decisions in local planning policy. Callway et al argue that health data could highlight local health priorities to developers and influence subsequent planning applications. This would be particularly beneficial for left behind communities who are often most negatively affected by poor development.

Indeed, our findings reveal significant levels of uncertainty and inconsistency amongst urban development actors about what constitutes healthy urban development. A senior Whitehall official indicated that, ‘the lack of a common view as to what [healthy] looks like allows people to kind of come up with their own interpretation’. Although there is a significant body of evidence demonstrating associations between the built environment and health and wellbeing outcomes (Bird et al., 2018), specific measures for incorporating that information into key decisions is lacking. By contrast, carbon accounting^2^ (National Highways, 2022) and biodiversity net gain (Natural England, 2023) are recognised measures that can be used to address climate change and the biodiversity impacts of urban development.

However, there is currently no widely recognised, simple to use metric that captures the many facets of healthy urban development. This lack of an easy metric makes it difficult to demonstrate the health impacts of urban development decisions. A local authority transport officer explained the point,

You’ve got this horrendous policy in the [National Planning Policy Framework] which says we can only refuse things if the impact is severe. Now define severe … I was asked this in a public inquiry … people living in high buildings, mental health issues associated with that, isolation, that’s very hard to quantify.

Subjective views on (un)healthy environments makes it difficult to build healthier urban areas, which can lock in inequalities for generations. This is particularly the case where decision makers, in the English discretionary planning system, feel unable to object to urban development proposals on health grounds due to the risk of expensive legal challenges (Montel, 2023). Robust and compelling evidence about the pathways between urban development and health outcomes are needed to support urban development actors in their decision-making. This includes epidemiological evidence demonstrating health impacts. However, it takes many years for health outcomes to be realised, requiring lengthy evaluation processes. In the short term, however, mechanisms and behaviours that are associated with health outcomes can be measured (e.g. physical activity levels). These insights can help to build a convincing evidence base for decision makers. Additional resourcing for monitoring and evaluation is needed, with partnerships between research and local government to develop an impactful evidence base that can help to address health inequalities (Black et al., 2021).

## Conclusions

The aim of this article has been to examine what needs to happen in central, sub-regional and local government to ‘level up’ public health in the UK. Overall, we advocate a long-term approach to preventative health. This is purported to have a much bigger impact on health inequalities than the individualistic, or ‘high agency’ interventions currently under-pinning the Government’s health missions in the Levelling Up White Paper (HM Government, 2022). By highlighting links between the urban environment and health, the wider determinants of health can be incorporated into the Government’s strategy for levelling up. This would ensure that the £4.8bn of levelling up funding, the bulk of which is allocated for infrastructure development, could also be used to benefit population health and tackle inequalities. Our seven recommendations in Table 4 detail what needs to happen in central and local government to achieve this.

Reducing health inequalities is a substantial task. However, hope can be found in past successful approaches. In England, evaluation of the National Health Inequality Strategy 1999–2010, implemented under the previous Labour Government, suggest that the strategy contributed to reductions in health inequalities on some measures (Holdroyd et al., 2022). Bambra (2022, p. 912) identified common themes for successfully tackling health inequalities across five international examples including the importance of ‘improving social and economic conditions through more expansive social policies’ alongside expanding access to health care and the political incorporation of groups in the population that suffer inequalities. This highlights how levelling up public health on a national scale is possible but must look beyond the healthcare system to include approaches to reduce economic inequalities.

Our findings make a timely contribution by placing public health at the centre of ongoing debates on Levelling Up within the social sciences. While our discussion has focussed on the challenges of levelling up health in the UK, health inequality is a global issue of significant importance. Moreover, cities around the world are struggling to improve the quality of their urban environments to create healthier places to live (Giles-Corti et al., 2022). As such, our recommendations on how to incorporate the wider determinants of health into the levelling up agenda have the potential for international relevance and traction, especially in countries characterised by highly centralised, Westminster models of administration.

A central theme to both the deep, structural causes of left behind areas and the potential solutions centre on the ability of Government to adopt a ‘whole of government’ approach to tackling health inequality (Meier et al., 2019). Breaking down silos and adopting a systems approach to policy development is critical to incorporating the wider determinants of health in meaningful ways. In this respect the academy must play its part. Scholars must continue to work across academic disciplines and fiefdoms to produce transdisciplinary evidence fit for purpose in a complex policy world. The social science traditions of esteemed scholars, such as Martin et al. (2021), have much to offer population health sciences, and vice versa. Our own transdisciplinary contribution in this field hopes to pave the way for future working across disciplines to address health inequalities in transformative ways.

## Supplementary Material

Supplementary Materials

## Figures and Tables

**Table 1:** Levelling up missions.

Mission	Description
1	By 2030, pay, employment and productivity will have risen in every area of the UK, with each containing a globally competitive city, and the gap between the top performing and other areas closing.
2	By 2030, domestic public investment in R&D outside the Greater Southeast will increase by at least 40%, and over the Spending Review period by at least one third. This additional government funding will seek to leverage at least twice as much private sector investment over the long term to stimulate innovation and productivity growth.
3	By 2030, local public transport connectivity across the country will be significantly closer to the standards of London, with improved services, simpler fares and integrated ticketing.
4	By 2030, the UK will have nationwide gigabit-capable broadband and 4G coverage, with 5G coverage for the majority of the population.
5	By 2030, the number of primary school children achieving the expected standard in reading, writing and maths will have significantly increased. In England, this will mean 90% of children will achieve the expected standard, and the percentage of children meeting the expected standard in the worst performing areas will have increased by over a third.
6	By 2030, the number of people successfully completing high-quality skills training will have significantly increased in every area of the UK. In England, this will lead to 200,000 more people successfully completing high-quality skills training annually, driven by 80,000 more people completing courses in the lowest skilled areas.
7	By 2030, the gap in Healthy Life Expectancy (HLE) between local areas where it is highest and lowest will have narrowed, and by 2035 HLE will rise by five years.
8	By 2030, well-being will have improved in every area of the UK, with the gap between top performing and other areas closing.
9	By 2030, pride in place, such as people’s satisfaction with their town centre and engagement in local culture and community, will have risen in every area of the UK, with the gap between top performing and other areas closing.
10	By 2030, renters will have a secure path to ownership with the number of first-time buyers increasing in all areas; and the government’s ambition is for the number of non-decent rented homes to have fallen by 50%, with the biggest improvements in the lowest performing areas
11	By 2030, homicide, serious violence and neighbourhood crime will have fallen, focused on the worst affected areas.
12	By 2030, every part of England that wants one will have a devolution deal with powers at or approaching the highest level of devolution and a simplified, long-term funding settlement.

Source: HM Government, 2022, pp. 6–7.

**Table 2:** Data collection teams.

Data gathering teams	Academic expertise
1	Local government
2	Urban development/health
3	Central government/health
4	Private developers/sustainability
5	Real estate
6	Management
7	Spatial and urban planning

**Table 3:** Profile of interview respondents.

Stakeholder primary role	Local/ Regional government	National government	Private sector	Other	Total
Property development	5	2	24	0	**31**
Urban planning	15	3	5	3	**26**
Finance	0	3	18	0	**21**
Transport	6	3	3	1	**13**
Public health	7	2		2	**11**
Politician	8	1	0	0	**9**
Environment/Sustainability	3	2	1	1	**7**
Other	5	4	2	3	**14**
**Total**	**49**	**20**	**53**	**10**	**132**

**Table 4:** What needs to happen to level up health?

	Martin et al.’s (2021) recommendations for levelling up	Recommendations for levelling up public health
1	Grasping the transformative moment for local, regional and urban development policy	Articulate a clear commitment to health prevention and tackling the wider determinants of health
2	Establishing a clear and binding national mission for levelling up	Health prevention must be designed as a cross-cutting agenda in Whitehall with clear accountability for delivery
3	Realising the potential of place in policymaking	Engaging with local actors, including members of the public, is critical to understanding local health needs
4	Decentralising towards a multilevel federal polity in the UK	Local government needs policy autonomy to adopt a systems approach to addressing health inequalities
5	Strengthening subnational funding and financing	Greater flexibility in local government funding is required to enable the integration of health with other policy areas
6	Embedding geography in the national state and policy machinery	Place sensitive approaches are essential for places that suffer the worst health outcomes
7	Improving subnational strategic research, intelligence, monitoring and evaluation capacity	Local health data needs to be utilised more effectively in urban development to highlight local health priorities

Source: Authors’ own.
