# Supplementary material for: What needs to happen to ‘level up’ public health?
Source: Contemp Soc Sci. Author manuscript; Available in PMC 2024 Jul 19. (PMC7616260; doi:10.1080/21582041.2023.2232765)
Supplement: Supplementary Materials [file EMS197385-supplement-Supplementary_Materials.pdf]

Wills, J. (2016). *Locating localism: Statecraft, citizenship and democracy*. Policy Press.
Wood, M., & Swift, J. (2022). *Levelling up the UK: What you need to know*. NHS Confederation.
World Health Organisation. (2015). *What you need to know about health in all policies*. Geneva: World Health Organization. Retrieved April 14, 2023, from https://cdn.who.int/media/docs/default-source/mca-documents/rmncah/health-in-all-policies-key-messages-en.pdf?sfvrsn=a4982d1_1

Appendix A

Study research questions

1. Who are the actors and how are actor networks structured?
2. What are the institutions that shape urban development and the context for decision-making?
3. How is power and influence structured in urban development actor networks?
4. What are actors' values, motivations and narratives?
5. How do actors perceive the problem (why healthy places are not created) in the urban development system and related to their activity and sphere of influence?
6. What is the process by which relevant decisions are made? To what extent are health outcomes (non-communicable diseases) and health inequalities being considered in decision making?
7. What resources (& evidence/tools) are available, and how are they (not) used in relation to actors' activity and sphere of influence?
8. How do actor networks function and what evidence and regulation do they need to deliver healthier development?

Appendix B

Coding framework

All data was coded during analysis into the following 23 categories. Each category contained multiple codes.

- Actor networks
- Barriers
- Characteristics of the urban development system
- Data relating directly to project case study sites
- Development type
- Economics
- Environmental sustainability
- Evidence
- Financial value
- Governance
- Health inequalities
- How health is included in decision-making
- Institutions
- Interventions
- Land
- Legal considerations
- Political considerations
- Power and influence
- Public involvement
- Risk
- Values
- What is needed to deliver healthier development
- Other
